# Intraguild Interactions between Egg Parasitoids: Window of Opportunity and Fitness Costs for a Facultative Hyperparasitoid

**DOI:** 10.1371/journal.pone.0064768

**Published:** 2013-05-21

**Authors:** Antonino Cusumano, Ezio Peri, Valentina Amodeo, Jeremy N. McNeil, Stefano Colazza

**Affiliations:** 1 Dipartimento di Scienze Agrarie e Forestali, Università degli Studi di Palermo, Palermo, Italy; 2 Department of Biology, The University of Western Ontario, London, Ontario, Canada; University of California, Berkeley, United States of America

## Abstract

We investigated intraguild interactions between two egg parasitoids of *Nezara viridula* (L.) (Heteroptera: Pentatomidae), *Ooencyrtus telenomicida* (Vassiliev) (Hymenoptera: Encyrtidae) and *Trissolcus basalis* (Wollaston) (Hymenoptera: Platygastridae), as the former has the potential to be a facultative hyperparasitoid of the latter. We assessed the suitability of *N. viridula* eggs for the development of *O. telenomicida* as a function of egg age when they were unparasitized, or had been attacked by *T. basalis* at different times prior to exposure to *O. telenomicida* females. *Ooencyrtus telenomicida* can exploit healthy *N. viridula* host eggs up to 5 days of age, just prior to the emergence of *N. viridula*. This window of opportunity can be extended for an additional 6–7 days through interspecific competition or facultative hyperparasitism. While there are minor fitness costs for *O. telenomicida* as the result of interspecific larval competition, those costs are greater with facultative hyperparasitism. In choice assays *O. telenomicida* females discriminated between different quality *N. viridula* eggs, avoiding those where their progeny would have to develop as facultative hyperparasitoids of *T. basalis*. Results are discussed with respect to the possible effects that the costs of intraguild parasitism might have on biological control programmes.

## Introduction

Intraguild interactions occur among organisms sharing a common resource [1] and “intraguild predation” (IGP), which is common in natural populations [2], occurs when two species that share a common host, under certain circumstances, prey upon each other [3]. Most IGP studies have focused on prey-predator interactions but recently it has been recognized that similar ecological interactions occur between host-parasitoid and host-pathogen interactions [4].

In parasitoid guilds there can be interspecific competitive interactions, either between adult parasitoids searching/exploiting hosts (extrinsic competition) or between parasitoid larvae developing within the same host (intrinsic competition) [5]–[7]. However, Rosenheim et al. [8] noted that intraguild parasitism can occur when one guild member is a facultative hyperparasitoid. Such species can act either as a primary parasitoid utilising some life stage of an herbivorous insect as a host, or as a hyperparasitoid where it uses a primary parasitoid as a host. Thus a facultative hyperparasitoid can exploit a healthy host but if it oviposits in a common host that has been already attacked by another species there are two possible outcomes: interspecific larval competition will occur if the competitor's offspring has not yet consumed all of the host resources, but if it has then hyperparasitism will occur [8], [9].

The evolution of facultative hyperparasitism is poorly understood [10] but may be key to the trophic shift from primary parasitism to obligatory hyperparasitism [11].

There are several documented cases of facultative hyperparasitism but this phenomenon is probably underestimated [10] and a real understanding of parasitoid trophic structure will only be achieved by very careful examination and dissection of host remains [12] and through the use of molecular techniques [13]. For example, *Trissolcus* spp. and *Ooencyrtus* spp. are parasitoids that exploit the eggs of the same stink bugs species and the latter group can develop as facultative hyperparasitoids of the former [14], [15]. Given that egg parasitoid guilds composed of *Ooencyrtus* and *Trissolcus* spp. have been reported in North America [16]–[19], South America [20], [21], Europe [22] and Japan [23], it is possible that both interspecific competition and facultative hyperparasitism occur and deserve to be investigated further.

This is not only important from a purely theoretical perspective, but also with respect to using parasitoids as biological control agents of important pests. There are benefits for a parasitoid that has the ability to be a facultative hyperparasitoid, such as an extended window of opportunity when it can successfully attack its host [24], [25], as well as gaining additional food resources [26]. However, there could also be associated fitness costs. It has been well documented that interspecific competition may result in longer development times, as well as smaller adults with reduced longevity and fecundity [27]: It is also possible that similar fitness costs may be associated with facultative hyperparasitism, due to the greater conversion costs when developing on entomophagous hosts [26], [28], [29].

While several studies have investigated intraguild predation [30]–[36], few have experimentally looked at intraguild parasitism [8], [37]–[40]. We, therefore, undertook a study to investigate interspecific interactions between *Trissolcus basalis* (Wollaston) (Hymenoptera: Platygastridae) and *Ooencyrtus telenomicida* (Vassiliev) (Hymenoptera: Encyrtidae), two idiobiont egg parasitoids of the Southern Green Stink Bug, *Nezara viridula* (L.) (Heteroptera: Pentatomidae) that co-occur in cultivated crops grown in Sicily. These parasitoid species differ in their host location and larval competitive abilities, with *T. basalis* being more efficient in host location [22], [41]–[43] while *O. telenomicida* largely dominates interspecific larval competition regardless of the order/time interval between oviposition events. Furthermore, *O. telenomicida* has the ability to develop as a facultative hyperparasitoid [15], [44].

We conducted experiments to determine: 1) the suitability of *N. viridula* eggs as a host for *O. telenomicida* as a function of time since they had been parasitized by *T. basalis* females; 2) the potential fitness costs, by comparing life history parameters of *O. telenomicida* when it developed in unparasitized *N. viridula* eggs, under interspecific competitive conditions (eggs containing a 1^st^ instar *T. basalis* larva) or as a facultative hyperparasitoid (where all host resources had been totally exploited by a mature *T. basalis* larva); 3) the preferences of *O. telenomicida* females when provided unparasitized *N. viridula* eggs, and host eggs previously exploited by *T. basalis* that would result in either interspecific competition or facultative hyperparasitism.

## Materials and Methods

### Insect rearing

The *Nezara viridula* colony, augmented regularly with field collected material, was maintained at 24±1°C, 70±5% RH, 16 h∶8 h L∶D on a diet of sunflower seeds and seasonal fresh vegetables that was changed every 2–3 days. All used insects were collected in the surroundings of Palermo, Italy. No specific permits were required for collection of insects. The collection sites were not privately owned or protected in any way and field samplings did not involve endangered or protected species.

Immatures and adults were kept in separate cages. Adult cages had paper towels as an ovipositional substrate and eggs were collected daily. The *O. telenomicida* and *T. basalis* colonies were established using wasps that emerged from naturally laid *N. viridula* egg masses or sentinel egg masses placed in the field. Colonies of each species were maintained at 24±2°C, 80±5% RH, 16 L∶8 D in 16-ml glass tubes and fed with a solution of honey–water. To maintain the colonies, newly laid *N. viridula* egg masses were exposed to five parasitoid females for 48 h, and the resulting male and female parasitoids were kept together to ensure mating. In all the bioassays 4–5 day old, mated females of *O. telenomicida* and *T. basalis* were used, and in all cases, parasitoids were naive with respect to oviposition. The wasps were isolated in small vials (1.5×5 cm) with a drop of honey–water solution one day before bioassays and transferred to the assay room at 24±1°C, 60±10% RH 1 h before being tested. Tests were conducted from 8:30 to 14:00 h and females were only used once.

### Bioassays

To test the window of opportunity of parasitism for *O. telenomicida* females a series of experiments was carried out. A female *O. telenomicida* was released at the center of a vertical, cylindrical Plexiglas® arena (diameter: 1.8 cm, height: 0.5 cm) with an egg mass (5 *N. viridula* eggs on a small piece of Parafilm®) located centrally on the floor. There were three different treatments: (I) unparasitized 1, 2, 3, 4 or 5 day old eggs; (II) 1 day old eggs parasitized by *T. basalis* and then exposed once to *O. telenomicida* 1, 2, 3, 4, 5, 6, 7 or 8 days later; and (III) 3 day old egg masses parasitized by *T. basalis* and then exposed once to *O. telenomicida* 1, 2, 3, 4, 5, 6, 7 or 8 days later. Each assay was observed and the *O. telenomicida* female was removed after she had parasitized all of the eggs. There were 10 replicates for each time interval of all three treatments and all egg masses held at 24±1°C, 70±5% RH, 16L∶8D so the number of *O. telenomicida* adults emerging from each egg mass could be recorded. The few host eggs that produced *T. basalis* adults or no parasitoid at all were not included in the subsequent analyses.

The possible effects of interspecific larval competition and facultative hyperparasitism on *O. telenomicida* were determined by comparing the number and sex ratio (% males) of emerging adults, as well as the developmental time, and size (estimated from the length of the hind tibia as done by Wajnberg et al. [45]) of both sexes when females were allowed to oviposit in host egg masses that were: (I) 1 day old and unparasitized (II) 2 or (III) 4 days old that had been parasitized by *T. basalis* 24 h earlier, or (IV) 10 days old that had been parasitized by *T. basalis* 7 days earlier. When *O. telenomicida* oviposited 24 h after *T. basalis*, the latter is at the stage of young 1^st^ instar larva but when *O. telenomicida* oviposited 7 days after *T. basalis*, the mature 3^rd^ instar larva of *T. basalis* has consumed all ooplasm and is ready to pupate. Thus treatments (II) and (III) represent natural situations of interspecific larval competition, while (IV) would be facultative hyperparasitism. Egg masses were held at 24±1°C, 70±5% RH, 16L∶8D and checked daily. Adults were frozen upon emergence (−18°C) then preserved in ethanol (70%) until the different measurements were taken.

Using the same experimental setup described above a choice bioassay was conducted to determine if *O. telenomicida* would exhibit an oviposition preference when simultaneously presented with different quality hosts. An *O. telenomicida* female was introduced in the arena containing a mass of 4 *N. viridula* eggs, one each of the following treatments: (I) a 1 day old unparasitized egg; (II) a 2 day old; and (III) a 4 day old egg that had been parasitized 24 h previously by *T. basalis*; and (IV) a 10 day old egg that had been parasitized 7 days previously by *T. basalis*. The oviposition preference was assessed in terms of “first oviposition”, *i.e.* the first host egg that has been parasitized by *O. telenomicida* under multiple choice conditions. There were 50 replicates and each was terminated after the *O. telenomicida* female had oviposited once.

### Statistical analysis

Data were tested for normality (Kolmogorov-Smirnov test) and if significantly different from a normal distribution were analyzed with non parametric tests. The effect of host age or time interval between oviposition by the two parasitoid species on the number of *O. telenomicida* adults that emerged, as well as the effect of different host quality on developmental time and hind tibia length were compared using a one-way ANOVA followed by Tukey test. The effect of host types on sex ratio was compared with the Kruskal-Wallis ANOVA and the Dunn test for multiple comparisons. The ability of *O. telenomicida* females to discriminate among hosts of different quality was tested with a χ^2^ test with Bonferroni correction. All statistical analyses were processed using STATISTICA7 software [46].

## Results

**Figure 1:**
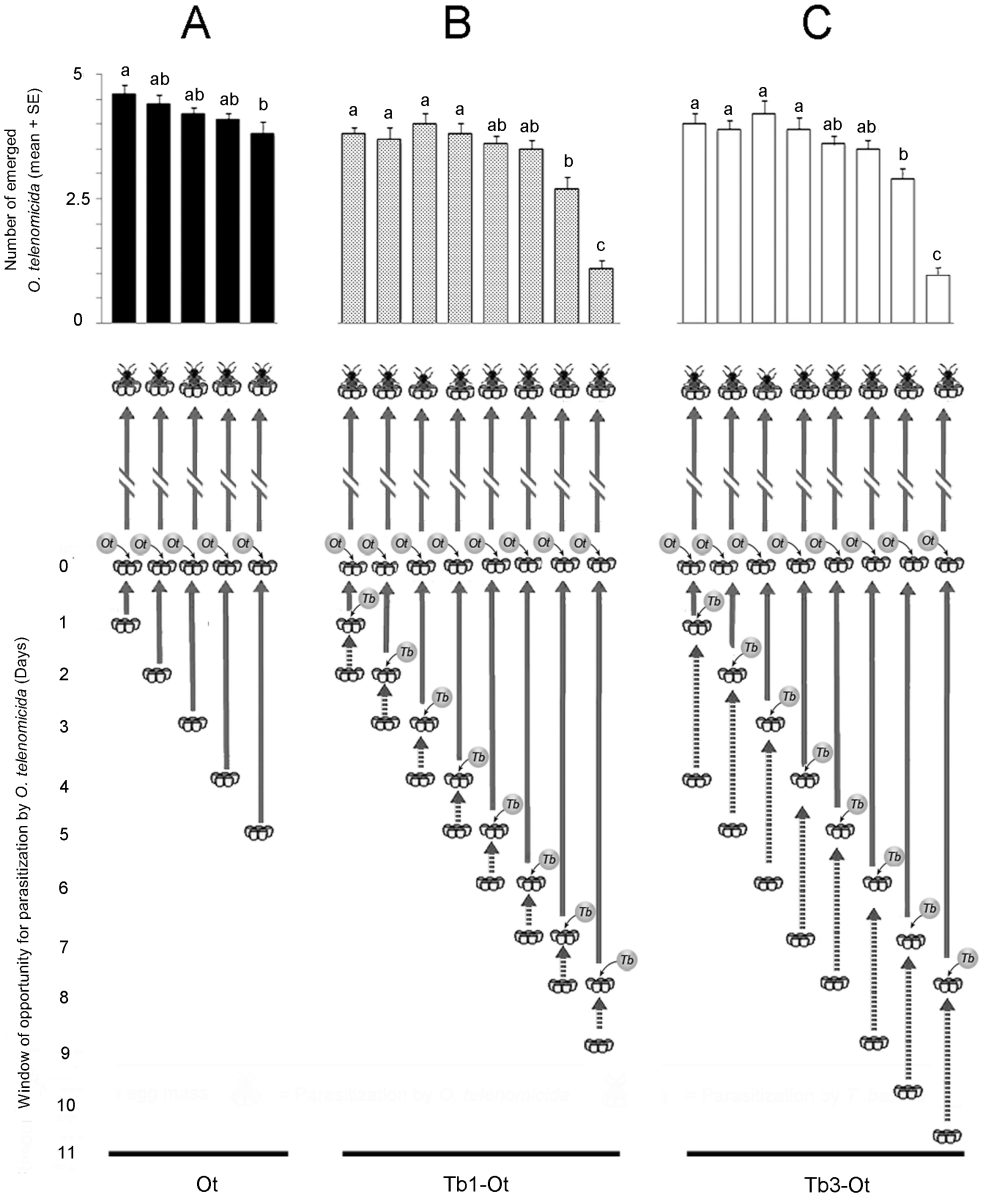
Window of opportunity for *Ooencyrtus telenomicida* as function of host egg age and interspecific parasitism status. The emergence of *Ooencyrtus telenomicida* from (A) unparasitized 1 to 5 day old *Nezara viridula* eggs (Ot); (B) 1day old *N. viridula* eggs parasitized by *Trissolcus basalis* that were then parasitized by *O. telenomicida* 1 to 8 days later (Tb1-Ot); and (C) 3 day old *N. viridula* eggs parasitized by *T. basalis* that were then parasitized by *O. telenomicida* 1 to 8 days later (Tb3-Ot).

There was a significant effect of host age on the number of adult *O. telenomicida* emerging from unparasitized *N. viridula* eggs (Fig. 1A; *F* = 3.21, *df* = 4, 45, *P*<0.05;), being significantly lower from 5 than from 1 day old hosts. Similar temporal effects were observed when *O. telenomicida* oviposited in *N. viridula* eggs that had been attacked by *T. basalis* when the eggs were 1 day old (Fig. 1B; *F* = 20.26, *df* = 7, 72, *P*<0.001) or 3 days old (Fig. 1C; *F* = 23.41, *df* = 7, 72, *P*<0.001. In both cases there was a decrease in the number of *O. telenomicida* emerging from the oldest hosts.

**Figure 2:**
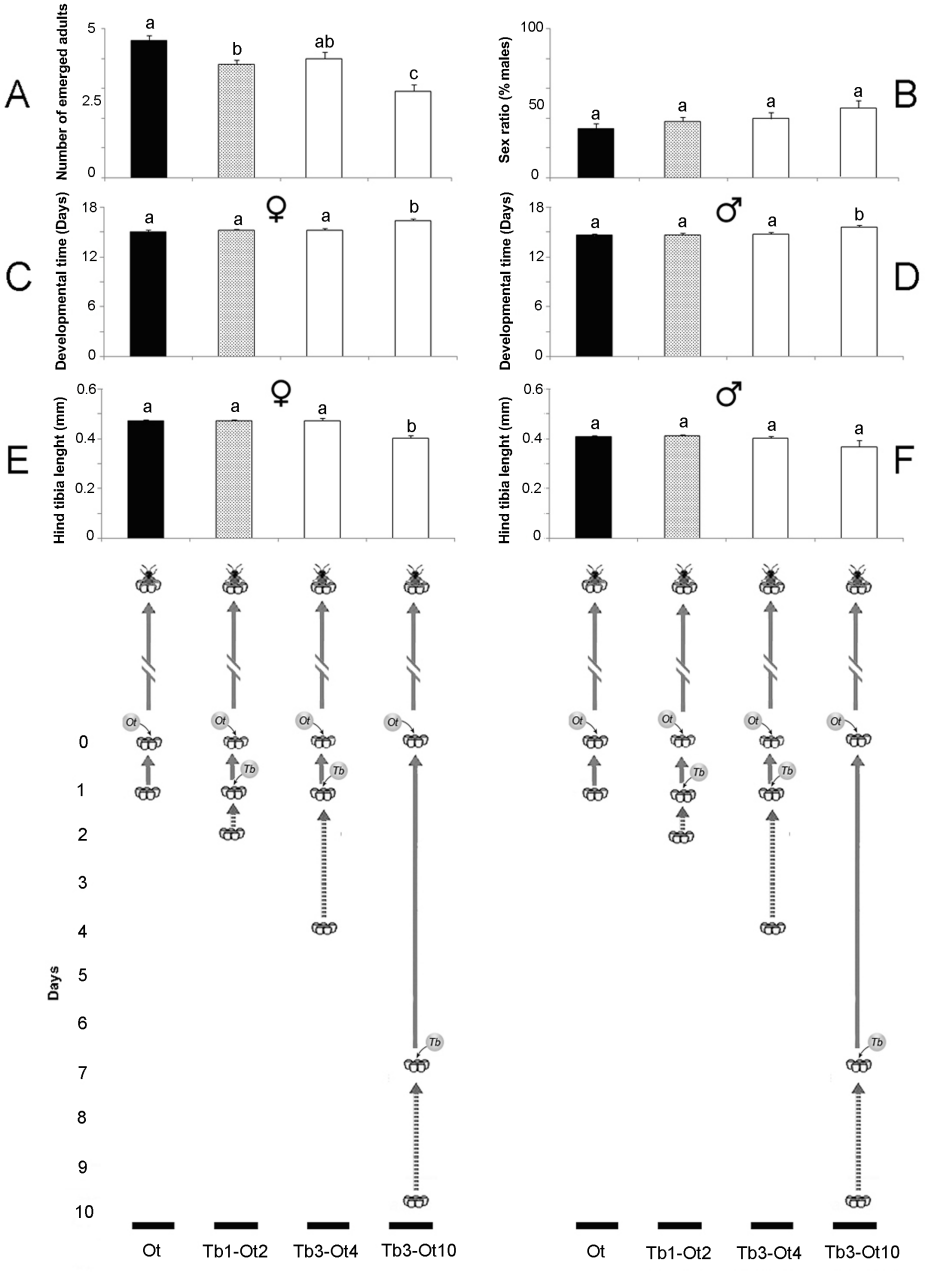
Life history parameters of *Ooencyrtus telenomicida* when developing in different host types. The number emerging (A), sex ratio (B), developmental time and size of female (C, E) and male (D, F) *Ooencyrtus telenomicida* adults developing in (I) 1 day old, unparasitized *Nezara viridula* eggs (Ot), (II) 2 day old *N. viridula* eggs that had been parasitized by *Trissolcus basalis* when they were 1 day old (Tb1 - Ot2), (III) 4 day old *N. viridula* eggs that had been parasitized by *T. basalis* when they were 3 days old (Tb3 - Ot4), or (IV) 10 day old *N. viridula* eggs that had been parasitized by *T. basalis* 7 days earlier (Tb3 - Ot10).

The average number of *O. telenomicida* adults produced was affected by the type of host exploited, (Fig. 2A; *F* = 13.84, *df* = 3, 36, *P*<0.001), generally being higher in previously unparasitized eggs than when in larval competition with, or as a facultative hyperparasitoid of *T. basalis*, although the proportion of males produced was similar in all treatments [Fig. 2B; *H*(3, N =  37) = 4.31, *P* = 0.229]. When *O. telenomicida* was a facultative hyperparasitoid of *T. basalis* the developmental time of both females (Fig. 2C; *F* = 20.67, *df* = 3, 36, *P*<0.001) and males (Fig. 2D; *F* = 5.51, *df* = 3, 33, *P*<0.001) was longer. Being a facultative hyperparasitoid also resulted in smaller females (Fig. 2E; F = 23.69, *df* = 3, 36, *P*<0.01), although male size was not affected (Fig. 2F; *F* = 2.53, *df* = 3, 33, *P* = 0.074).

*Ooencyrtus telenomicida* females clearly discriminated between the different host egg types, avoiding host eggs that contained well developed *T. basalis* larvae where they would have to develop as a facultative hyperparasitoid (Table 1; *χ* ^2^ = 17.68, *df* = 3 *P*<0.001). Interestingly, there was a marginal preference for eggs that had been attacked by *T. basalis* when they were 1 day old over unparasitized eggs (*χ* ^2^ = 3.46, *df* = 1, *P* = 0.06), or those attacked by *T. basalis* when they were 3 days old (*χ* ^2^ = 2.78. *df* = 1, *P* = 0.09).

**Table 1:** The proportion of *Ooencyrtus telenomicida* females selecting a (I) 1 day old, unparasitized *Nezara viridula* eggs (Ot), (II) 2 day old *N. viridula* eggs that had been parasitized by *Trissolcus basalis* when they were 1 day old (Tb1 - Ot2), (III) 4 day old *N. viridula* eggs that had been parasitized by *T. basalis* when they were 3 days old (Tb3 - Ot4), or (IV) 10 day old *N. viridula* eggs that had been parasitized by *T. basalis* 7 days earlier (Tb3 - Ot10) as their first oviposition site in a choice bioassay.

*O. telenomicida* ovipositing in *N. viridula* egg mass assembled using 4 different egg types				
Egg types	Ot	Tb1-Ot2	Tb3-Ot4	Tb3-Ot10
**Egg age**	1	2	4	10
**Egg age when parasitized by Tb**	-	1	3	3
**Choice (%± SE)**	24.0±6.0 a	46.0±7.1 a	26.0±6.2 a	4.0±2.8 b

## Discussion

In Sicily, more *T. basalis* adults emerge from parasitized field-collected *N. viridula* eggs than *O. telenomicida*, (Cusumano personal observations), which is not particularly surprising given the superior abilities of the former to locate suitable hosts [22]. Females of both *T. basalis* and *O. telenomicida* exploit volatile cues emitted by *N. viridula* virgin males and pre-ovipositing females [22], [41]. In addition, *T. basalis* females use contact kairomones in host footprints and volatile oviposition-induced synomones [22], [41], [42], [47]–[49], so foraging females not only utilize more cues than *O. telenomicida*, but also ones that are more reliable indicators of the presence of host eggs. Furthermore, *T. basalis* females also have a higher total lifetime fecundity than *O. telenomicida* so the chances that *O. telenomicida* females find unparasitized egg masses may be quite low under field conditions.

However, as seen from the results of this study, *O. telenomicida* has evolved several strategies that increase the window of opportunity to exploit host eggs. For example, *N. viridula* eggs hatch after 5 days under our laboratory conditions and while *T. basalis* can only successfully develop on unparasitized *N. viridula* eggs that are <4 days old [50], *O. telenomicida* successfully exploits unparasitized *N. viridula* eggs up to the time of host emergence (Fig. 1A), similar to the congeneric, *O. nezarae* Ishii, an egg parasitoid of the bean bug *Riptortus clavatus* Thunberg (Heteroptera: Alydidae) [51]. Furthermore, *O. telenomicida* is clearly superior under the conditions of interspecific larval competition, whether the eggs that have been attacked by *T. basalis* were 1 or 3 days old (Fig. 1), as in all of our experiments, <15% of all parasitoid adults were *T. basalis*. In addition, when acting as facultative hyperparasitoid (Fig. 1c), *O. telenomicida* can effectively exploit eggs for at least 10 days after they are laid by *N. viridula* females.

There are fitness costs for *O. telenomicida*, associated with both interspecific competition and facultative hyperparasitism (Fig. 2). In the case of competition the only significant effect observed was a lower number of *O. telenomicida* adults emerging when there was early-stage interspecific larval competition (Fig. 2A). Interestingly, in the choice bioassays, *O. telenomicida* showed a marginally significant preference for 2 day old eggs recently parasitized by *T. basalis*, over unparasitized ones and 4 day old eggs that *T. basalis* had attacked 1 day earlier, even though fewer adults emerged (Table 1, Fig. 2A). At oviposition, *T. basalis* injects substances that arrest embryonic development of the host and when the parasitoid's egg hatches teratocytes are released that alter the ooplasm [52]. To what extent these two events associated with the development of *T. basalis* affects the suitability of the eggs for *O. telenomicida*, when interspecific competition occurs, remains to be clarified.

In the case of facultative hyperparasitism the development time of both sexes was longer and females were significantly smaller (Fig. 2). This could be important as adult body size has been correlated with survival and reproductive success in many parasitoid species [11], [53], [54] although, as seen in the choice bioassays, *O. telenomicida* females will avoid hosts that result in facultative hyperparasitism if a choice is available (Table 1). If certain conditions resulted in high levels of facultative hyperparasitism this could impact on subsequent population dynamics at all trophic levels, and affect the efficacy of biological control programmes. As pointed out by Boivin and Brodeur [29], assessing the impact of a species that act simultaneously as primary parasitoid, interspecific competitor and facultative hyperparasitoid is a huge challenge, both theoretically and experimentally. However, the few studies examining the potential fitness costs of facultative hyperparasitsm have come up with quite varied findings, some showing there are fitness costs [26], [55], while others have found few or no effect [56], [57]. Therefore, it is clear that in order to understand the potential tradeoffs between the benefits accrued by a species that has the potential to be a facultative hyperparasitoid and the potential negative effects on all parasitoid species in the guild, both from basic and applied perspectives, considerably more information must be gathered from systems where interguild parasitism exists.
